# Comparing the effects of different electromagnetic stimulation on lower limb motor impairment after stroke: a protocol for systematic review and network meta-analysis

**DOI:** 10.3389/fneur.2026.1828469

**Published:** 2026-05-29

**Authors:** Raoting Zhang, Song Li, Shaoyan Yao, Zhihong Chen, Yu Xu, Yanbei Chen, Xiaoju Zhu

**Affiliations:** 1 Qujing Hospital of Traditional Chinese Medicine, Qujing, China; 2 The Second Clinical Medical College, Heilongjiang University of Chinese Medicine, Harbin, China; 3 Yunnan University of Chinese Medicine, Kunming, China

**Keywords:** lower extremity, motor impairment, network meta-analysis, protocol, randomized controlled trial, stroke

## Abstract

**Objective:**

Stroke is a leading cause of death and long-term disability worldwide. Most survivors develop multiple functional impairments, with lower limb dysfunction being particularly prevalent and impactful, and has emerged as a key factor undermining patients’ quality of life. Electromagnetic stimulation therapy, a potential intervention for post-stroke lower limb rehabilitation, remains controversial regarding its clinical efficacy in current literature. Furthermore, no definitive conclusions exist regarding the comparative advantages of distinct electromagnetic stimulation protocols. Given these controversies and uncertainties, this study aims to perform a network meta-analysis (NMA) to systematically assess the effects of distinct electromagnetic stimulation protocols on post-stroke lower limb motor function. This analysis further aims to quantify differences in clinical benefits across these protocols and characterize their safety profiles, thereby offering evidence-based guidance for clinical decision-making.

**Methods:**

We will conduct a systematic search of the following eight databases: PubMed, Embase, the Cochrane Library, Web of Science, CNKI, the VIP Database, the Wanfang Database, and the China Biomedical Literature Database. The search will encompass randomized controlled trials published from the inception of each database until February 15, 2025. Subsequently, two independent reviewers will assess the risk of bias for all included studies using the Cochrane Risk of Bias tool (RoB 2). We will then perform an NMA using a random-effects model in Stata software to compare the efficacy and safety of various electrical stimulation therapies. The surface under the cumulative ranking curve (SUCRA) will be calculated to estimate the comparative benefits and harms of each intervention.

**Discussion:**

This evaluation protocol aims to generate evidence on the efficacy of various electromagnetic stimulation regimens for improving clinical symptoms in patients with post-stroke lower limb dysfunction. This evidence will thereby provide a theoretical foundation and practical insights to aid clinicians in optimizing diagnostic and therapeutic decision-making.

**Clinical trial registration:**

https://www.crd.york.ac.uk/, identifier [CRD420251247508].

## 1 Introduction

Stroke is a severe cerebrovascular disease and currently stands as one of the leading causes of death and disability in adults globally (1). Although medical advances have reduced the mortality rate of stroke (2), approximately 90% of survivors still suffer from motor dysfunction of varying degrees (3). Among these survivors, approximately 60% suffer from lower limb impairments, such as the loss of independent walking ability (4–6). Such impairments not only severely affect the daily lives of patients but also impose substantial psychological and financial burdens on patients and their families (6). Therefore, exploring scientific, effective, and safe rehabilitation approaches is crucial for the economic and psychological well-being of post-stroke patients with lower limb dysfunction and their families.

Rehabilitation strategies for post-stroke lower limb dysfunction predominantly include functional training and physical stimulation. Clinically common functional training modalities include virtual reality (7), occupational therapy, exercise therapy (8), and robot-assisted training (9). As a core component of such functional training modalities, task-oriented activities promote neural remodeling, thereby facilitating long-term functional recovery. However, their effectiveness is often limited by patient adherence, cost, and therapeutic onset rate. In contrast, physical stimulation exerts a direct effect on neuromuscular tissues, triggering rapid physiological responses. Physical stimulation exhibits a faster therapeutic onset and higher specificity, and is often combined with functional training to synergistically enhance therapeutic outcomes.

Electromagnetic stimulation is a physical intervention method that acts on nerve or muscle tissue via electric or magnetic fields to regulate neural excitability or muscle contraction. It is a type of physical intervention widely used in post-stroke rehabilitation (10). In recent years, numerous clinical trials have confirmed that electromagnetic stimulation exerts a notable ameliorative effect on post-stroke lower limb dysfunction (11–13). Currently, the commonly used electromagnetic stimulation methods for treating lower limb dysfunction following stroke primarily include central nervous system stimulation techniques, such as repetitive transcranial magnetic stimulation (rTMS) (14), intermittent theta burst stimulation (iTBS) (15) and transcranial direct current stimulation (tDCS) (16); They also include peripheral nerve or muscle stimulation techniques, such as neuromuscular electrical stimulation (NMES) (17) and functional electrical stimulation (FES) (18). In addition to these electromagnetic stimulation techniques, acupuncture, a traditional Chinese medical therapy, is currently recommended by the World Health Organization (WHO) as a common complementary rehabilitation intervention for post-stroke recovery (19). With the advancement of medical technology, researchers have integrated electrical stimulation with acupuncture to develop transcutaneous electrical acupuncture stimulation (TEAS) and electroacupuncture (EA), both of which incorporate the characteristics of electrical stimulation and acupuncture. These techniques have also been shown to enhance lower limb function in stroke patients (20, 21). Mechanistically, regarding electromagnetic stimulation, extensive research confirms that it promotes neural remodeling and the reorganization of motor function by regulating neuronal excitability, inducing synaptic plasticity, and promoting the expression of neurotrophic factors (22–24). It regulates the excitability balance between cerebral hemispheres, restores the function of the motor cortex-spinal cord pathway, thereby improving motor control, reducing spasticity, and facilitating motor recovery (25).

Existing studies predominantly use paired meta-analyses to investigate differences in efficacy among various electromagnetic stimulation therapies for post-stroke lower limb dysfunction. However, these analyses are confined to direct comparisons of only two interventions and fail to provide comprehensive assessments of multiple electromagnetic stimulation protocols (20, 26–28). In contrast, network meta-analysis (NMA) synthesizes both direct and indirect evidence from relevant studies. Through this integration, it synthesizes data from multiple randomized controlled trials (RCTs), assesses the relative efficacy of various interventions, and lays a scientific foundation for identifying optimal treatment strategies for patients. This comprehensiveness offers more robust evidential support for the systematic comparison of different treatment protocols.

Two NMA studies have evaluated the efficacy of 10 types of electromagnetic stimulation therapies for post-stroke lower limb dysfunction (11, 13). However, these studies focused solely on electromagnetic or magnetic stimulation therapies in isolation and did not directly compare these two intervention modalities. Furthermore, its inclusion of electromagnetic therapies is limited, as it excludes commonly used electromagnetic stimulation methods such as TENS and EA. These limitations may compromise the comprehensiveness and clinical applicability of their findings. Therefore, based on published RCTs, this study aims to comprehensively compare the efficacy and safety of different electromagnetic stimulation protocols for post-stroke lower limb dysfunction, with the goal of providing more reliable and comprehensive evidence for research and clinical practice.

## 2 Materials and methods

### 2.1 Study registration

The design and reporting of this systematic review and NMA protocol will strictly comply with the preferred reporting items for systematic reviews and meta-analyses protocols (PRISMA-P) guideline (29) and the PRISMA extension statement for network meta-analyses (30). Moreover, this study protocol has been prospectively registered on the international prospective register of systematic reviews (PROSPERO), with the registration number CRD420251247508. Meanwhile, the full PRISMA-P checklist is provided in Supplementary file S1.

### 2.2 Eligibility criteria

This systematic review will perform literature screening based on the PICOS framework, as follows.

#### 2.2.1 Types of studies

This study will only include RCTs published in peer-reviewed journals, with no restrictions on language or geographic region. Non-randomized controlled trials, case–control studies, cohort studies, case reports, and other similar study designs will be excluded.

#### 2.2.2 Types of participants

On the premise of confirming the study type, participants in this study must meet internationally recognized diagnostic criteria for stroke (including ischemic and hemorrhagic subtypes) and present with unilateral or bilateral lower limb dysfunction. Individuals with lower limb dysfunction attributable to etiologies other than stroke will be excluded. Additionally, this study imposes no restrictions on participants’ age, gender, stroke severity, lesion location (e.g., left or right cerebral hemisphere), or disease duration.

#### 2.2.3 Types of interventions

After determining the study population, all participants in the intervention group will receive neurostimulation interventions comprising two main categories of electromagnetic stimulation techniques: central nervous system stimulation techniques, such as rTMS, iTBS, and tDCS; and peripheral nerve or muscle stimulation techniques, such as NMES, EA, TEAS, and FES. Meanwhile, the intervention group may be combined with other non-electromagnetic rehabilitation methods recommended by relevant authoritative guidelines (31), such as physical therapy, occupational therapy, robot-assisted rehabilitation, and conventional pharmacological treatments for stroke.

### 2.3 Types of control groups

This study includes all literature in which the control group received either guideline- recommended standard care (31) or commonly used clinical rehabilitation interventions. Studies involving the administration of any type of electromagnetic stimulation therapy to participants will be excluded. Based on the aforementioned inclusion and exclusion criteria, the control group interventions will be stratified into three levels as necessary to reduce heterogeneity and ensure analytical validity, as specified below: (1) Sham stimulation group: Consists of sham biological or physical therapies; (2) Conventional medication group for stroke patients: Refers to routine pharmaceutical interventions consistent with clinical practice; (3) Active intervention group: Denotes interventions that go beyond conventional treatments and have proven efficacy (e.g., physical therapy, occupational therapy, robot-assisted rehabilitation).

### 2.4 Types of outcomes

#### 2.4.1 Primary outcomes

This study will employ the Fugl-Meyer Lower Extremity Functional Assessment (FMA-LE) as the primary outcome measure. Specifically, the FMA-LE is a widely recommended, reliable scale for assessing post-stroke lower limb motor impairment, acting as a robust tool for comprehensive evaluation of motor function. Notably, it exhibits excellent predictability, reliability, and sensitivity for lower limb motor recovery in stroke patients (32, 33). Comprising 17 items (each scored 0–2, total score 34), higher scores on the scale indicate superior lower limb motor function.

#### 2.4.2 Secondary outcomes

This study will utilize the 10-meter walk test (10MWT) (34), which assesses short-distance walking speed and is a key indicator of limb motor function, along with the Berg Balance Scale (BBS) (35) to measure overall balance ability. Additionally, assessment tools focusing on activities of daily living (ADL) capability, such as the modified Barthel Index (MBI) (36), will serve as secondary outcome measures in this research. Safety outcomes primarily refer to adverse events (AEs), including clinical symptoms such as palpitations, nausea, and vomiting, which will be evaluated using a ternary scoring system (0 = absent, 1 = present). When necessary, pooled analysis of AE frequency will be further performed according to severity grading: mild (minimal discomfort requiring no treatment), moderate (discomfort relieved by over-the-counter medication), and severe (conditions requiring urgent clinical intervention or discontinuation of treatment).

### 2.5 Data source and search strategy

This study will adopt a rigorously designed literature retrieval strategy to ensure comprehensive coverage of relevant studies. To balance the precision and comprehensiveness of retrieval results, a dual-search approach that combines Medical Subject Headings (MeSH) and free-text terms will be adopted. Detailed retrieval strategies for Chinese and English databases are provided in Supplementary file S2.

Meanwhile, the retrieval protocol, developed based on Boolean logic, will cover all accessible literature in each database from its inception to February 15, 2025. Two reviewers with systematic training will independently perform the retrieval in eight Chinese and English databases, including PubMed, Embase, Cochrane Library, Web of Science, China National Knowledge Infrastructure (CNKI), VIP Database, Wanfang Database, and China Biomedical Literature Database (CBM). Additionally, we will conduct manual searches and screen relevant review articles and reference lists to identify other eligible studies.

### 2.6 Study selection

**Figure 1:**
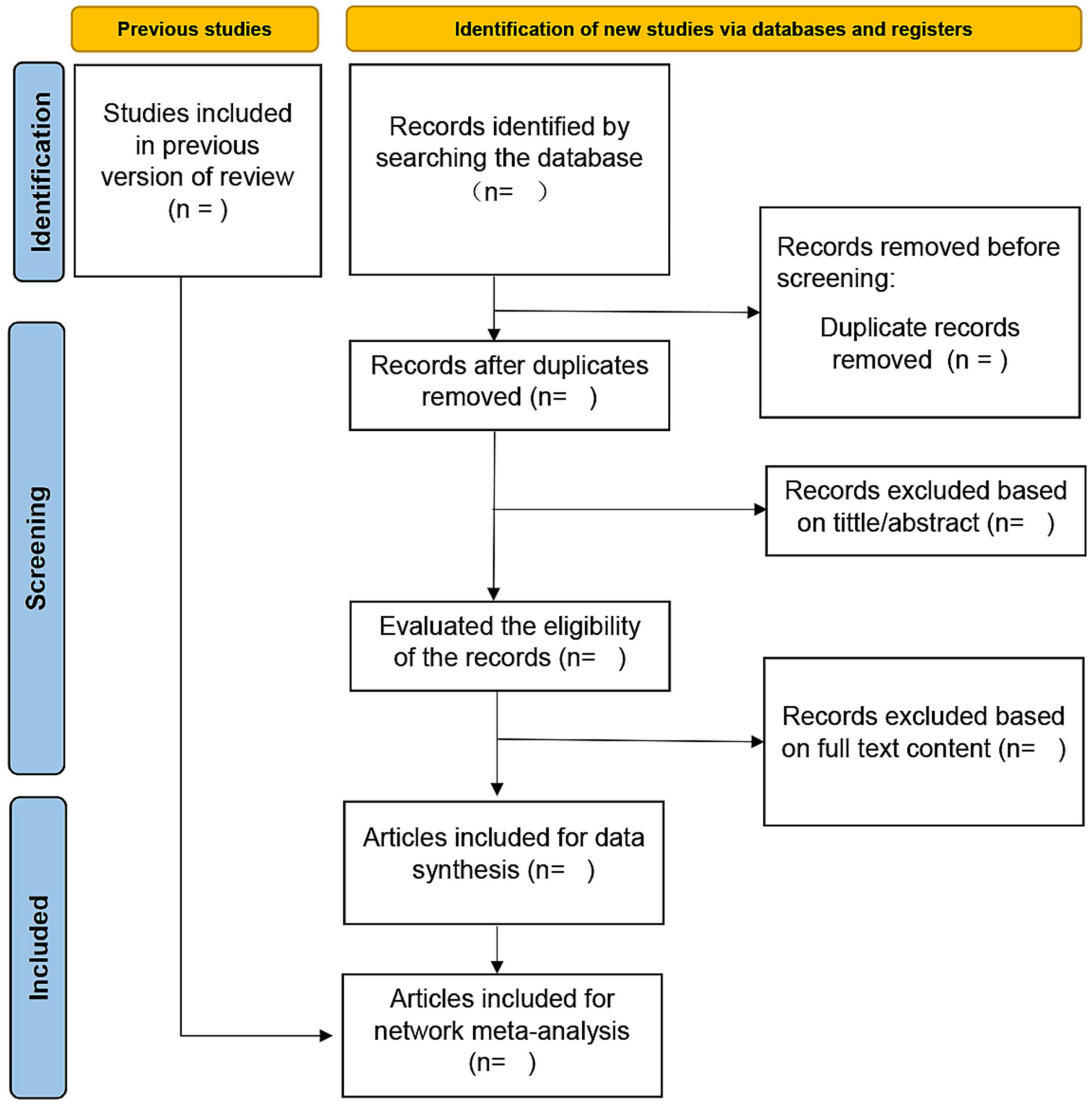
Flow diagram of study selection process. (*n*) is the number of articles that will be included at each stage.

Two reviewers will independently perform literature searches. First, they will use EndNote X9 software to remove duplicate records; this step will then be followed by manual verification to ensure thorough deduplication. After deduplication, an initial screening will be performed based on titles and abstracts, followed by a full-text review. Throughout the screening process, predefined inclusion and exclusion criteria will be strictly followed, thereby ensuring the validity of the selected literature and the reliability of the final study cohort. After both reviewers complete the above screening, they will cross-verify their respective results. Any discrepancies arising during this cross-verification will be resolved by a third-party expert reviewer. The PRISMA flow diagram (Figure 1) fully illustrates the entire process of literature searching and screening in this study.

### 2.7 Data extraction

In this systematic review, two independent reviewers will perform systematic data extraction using a pre-developed Excel template to retrieve relevant information from the studies included in the final analysis. The extracted data set will cover the following domains: (1) Study characteristics: authors, publication year, country, study design, and research center; (2) Participant characteristics: sample size, gender, age, diagnostic criteria, disease status (duration, infarct location, paralyzed limb(s)), medication use, treatment duration, and follow-up procedures; (3) Intervention and control details: type of electromagnetic, duration, frequency, treatment protocol, and key stimulation parameters; (4) Outcome measures: all quantitative assessments, records of adverse events, and measurement time points.

This study will utilize all continuous data for calculating pre- to post-treatment value changes—specifically, the differences in pre- to post-treatment outcome indicators. If these values are not reported in the original studies, they will be derived using the following method, with the correlation coefficient (corr) typically set to 0.5.


$$\begin{array}{l} {\mathrm{SD}}_{E}\;\text{change}=\\ \sqrt{{\mathrm{SD}}_{E\;\text{baseline}}^{2}+{\mathrm{SD}}_{E\;\text{final}}^{2}-(2\times \text{Corr}\;{\mathrm{SD}}_{E}\;\text{baseline}\times {\mathrm{SD}}_{E}\;\text{final})} \end{array}$$

$$\mathrm{Mea}n_{E\;\text{change}}=\mathrm{Mea}n_{E\;\text{final}}-\mathrm{Mea}n_{E\;\text{baseline}}$$

When research evaluation results reported in the literature are inconsistent, we will conduct data conversion and standardization. The study will set 4 weeks of treatment as the standard time point for outcome assessment; for studies that do not directly report 4-week follow-up data, estimates will be derived using linear interpolation (for continuous outcomes) or proportion approximation (for binary outcomes). Additionally, any discrepancies arising during the data extraction process will be resolved through arbitration by a third senior reviewer. For instances involving incomplete or ambiguously presented data, we will contact the corresponding authors via email to request complete and detailed data. For multi-arm studies, in order to prevent their correlations from affecting the overall analysis, we will split or combine the data in accordance with the guidelines. Finally, all data will undergo cross-verification prior to statistical analysis; any discrepancies identified during this process will be discussed within the review panel to reach a consensus on the final data.

### 2.8 Risk of bias assessment

This systematic review will involve two reviewers conducting independent risk-of-bias assessments of the included randomised controlled trials using the Cochrane Risk of Bias 2.0 tool (RoB 2) (37). Preceding formal assessment, both reviewers will undergo standardised methodological training and strictly adhere to detailed operational guidelines to minimise bias arising from subjective judgment. The RoB 2 tool conducts assessments across five core dimensions: (1) Randomisation process; (2) Deviations from intended interventions; (3) Missing outcome data; (4) Measurement of the outcome; (5) Selection of the reported result. For each predefined outcome measure, the reviewers will assess the included studies across these dimensions as having a low risk of bias, a moderate risk of bias, or a high risk of bias. In the event of a disagreement between two reviewers, consensus will first be sought through in-depth discussion; if necessary, the matter will be referred to a third reviewer for adjudication. The overall RoB 2 assessment results for all studies and outcomes will be collated and presented visually using summary risk of bias plots and statistical charts.

The results of the risk of bias assessment will be incorporated into subsequent meta-analyses: studies assessed as having a high risk of bias will be excluded from the main meta-analysis where appropriate; sensitivity analyses will also be conducted using only studies with a low risk of bias. Furthermore, the overall risk of bias rating will be used to downgrade the quality of evidence within the GRADE (Grading of Recommendations, Assessment, Development and Evaluation) system.

### 2.9 Data synthesis

#### 2.9.1 Assessment of transitivity

The transitivity assumption is a core prerequisite for the validity of network meta-analysis results. To rigorously verify and ensure the validity of this assumption, this study pre-specified the stage of stroke onset, stroke type, baseline severity of neurological deficits, electromagnetic stimulation parameters (such as stimulation intensity and frequency), duration of intervention, and the presence or absence of other adjunctive rehabilitation therapies as key potential effect modifiers. Descriptive statistical methods will be employed to compare whether there is significant clinical heterogeneity among these factors across different centres, thereby verifying the robustness of the transferability assumption.

#### 2.9.2 Pairwise meta-analysis

In this systematic review, we will conduct statistical analyses using STATA 18.0 software, serving as the core tool for subsequent meta-analytic procedures. For direct evidence pertaining to primary outcome measures, we will conduct a paired meta-analysis adopting a random-effects model to compare the clinical efficacy of various electromagnetic stimulation regimens versus control interventions. The primary outcome measure of the included studies is the FMA-LE score (a continuous variable). Accordingly, the standardized mean difference (SMD) and its 95% confidence interval (95% CI) will be used as the pooled effect measure, with statistical significance defined as *p* < 0.05.

#### 2.9.3 Network meta-analysis

For the network meta-analysis, we will select a random-effects model based on a frequency framework to analyze both direct and indirect evidence across all interventions. Specifically, indirect comparisons will be conducted via a common comparator C under the assumption of transitivity, where the relative effect between intervention A and intervention B is estimated from the known effects of A versus C and B versus C. The consistency assumption (i.e., the agreement between direct and indirect evidence for the same comparison) will be formally evaluated using the inconsistency assessment methods described below. Heterogeneity will be assessed using the I^2^ statistic and, if sufficient data are available, explored through subgroup or meta-regression analyses.

This study will employ a combined global and local approach to assess inconsistencies within the network. Global inconsistencies will be tested using a design-treatment interaction model, with *p* < 0.05 as the threshold for significant inconsistency; local inconsistencies will be assessed using the node splitting method, calculating the differences in effect estimates for direct and indirect comparisons, respectively, and using the criterion of whether the 95% confidence interval contains 0 or a *p*-value < 0.05 from the loop-specific test. If significant inconsistencies are detected, their potential sources will first be explored from the perspectives of clinical and methodological heterogeneity, and sensitivity analyses will be conducted to assess their impact on the robustness of the conclusions; if the inconsistencies contradict clinical information or cannot be reasonably explained, the relevant comparative results will be interpreted only on an exploratory basis.

For continuous outcomes, the pooled SMD or mean difference (MD) will be adopted to calculate the corresponding 95% CI. For dichotomous outcomes, the pooled risk ratio (RR) will be estimated with the corresponding 95% CI. The data analysis workflow is as follows: First, the “networkplot” command will generate a network evidence diagram to visually quantify the association between studies and interventions. Nodes represent distinct interventions, edges denote pairwise comparisons between interventions, and node size is proportional to sample size. Subsequently, the study will employ “mvmeta inconsistency” for global inconsistency testing to assess the consistency between direct and indirect comparison evidence. If closed loops exist in the network diagram, we will implement node splitting via the “network sidesplit all, tau” command to further examine inconsistencies. Building on this, a random-effects model of NMA will synthesize data to compare the relative efficacy of different electromagnetic therapies. Subsequently, the “SUCRA” command will rank the effects of each intervention and generate a cumulative probability plot. The surface under the cumulative ranking curve (SUCRA) reflects the relative superiority of interventions, with higher SUCRA scores indicating better intervention efficacy. Finally, the “netfunnel” command generates funnel plots to visually assess publication bias and small-sample effects in the included studies.

### 2.10 Sensitivity analysis

This study will perform sensitivity analyses to validate the robustness and reliability of the NMA results, identify potential bias-inducing factors, exclude studies with a higher risk of bias, and define the stability boundaries of the final conclusions. This approach will yield more reliable evidence to support clinical decision-making.

### 2.11 Subgroup analysis

This study will stratify participants according to intervention type (central stimulation, peripheral stimulation and electroacupuncture), stroke phase (acute, subacute and recovery), duration of follow-up, and the severity of baseline neurological deficits. Where data permit, we will further categorise interventions based on key stimulation parameters (including stimulation frequency, current intensity, pulse width, cortical target, duration of a single treatment session, and total number of treatment sessions). For interventions with significant differences in stimulation parameters, stratified analysis will enable us to effectively control for heterogeneity between groups, enhance the relevance and reliability of the study results, and ensure the robustness of the generalisability assumption in the network meta-analysis.

### 2.12 Assessment of reporting bias

This systematic review will utilize funnel plots to detect potential reporting bias for outcomes with at least 10 included studies. As a visual tool for bias identification, funnel plots are constructed such that the y-axis represents effect size and the x-axis denotes the standard error (SE) of the effect size. Essentially, they are employed to investigate reporting bias and issues related to small effect sizes (38). Typically, this analysis focuses on the comparison nodes of individual interventions within the network structure. Ideally, the included studies should show a symmetrical distribution in the plot, whereas significant asymmetry indicates potential publication bias. Furthermore, for dichotomous outcomes, calibrating effect sizes using the “inverse variance method” prior to plotting improves the accuracy of the analysis. In conclusion, funnel plots act as a reliable visual tool for identifying publication bias and other forms of bias in systematic reviews.

### 2.13 Grading the strength of evidence

Two independent reviewers will assess this study using the Grading of Recommendations, Assessment, Development and Evaluation (GRADE) framework (39). Specifically, the certainty of each outcome will be systematically evaluated by synthesizing the combined and individual effect sizes of each outcome measure and considering the overall certainty of the evidence. Any discrepancies arising between the two reviewers will be resolved through consultation with a third independent reviewer. Additionally, GRADE evidence quality will be downgraded if any of the following factors are present: risk of bias, inconsistency in effect sizes, indirectness of evidence, imprecision of results, and publication bias. This framework classifies the strength of evidence into four levels, as follows: high, moderate, low, and very low. Among these levels, high-quality evidence indicates that the true effect is highly consistent with the estimated effect; consequently, such evidence warrants strong recommendations for clinical decision-making. By contrast, very low-quality evidence indicates that the true effect is likely to deviate substantially from the estimated effect.

### 2.14 Ethics and dissemination

The data of this study were derived from previously published RCTs. The research process involved neither direct contact with patients or the public nor the use of personal private information, thus avoiding infringement of any relevant rights and interests. Based on the above, this study did not require ethical review and approval. Additionally, the findings of this study will, following rigorous peer review, be published in relevant academic journals and disseminated through compliant academic channels.

## 3 Discussion

Despite the declining mortality rate of stroke, approximately 60% of survivors continue to suffer from lower limb dysfunction (e.g., inability to walk independently) after stroke onset, which results in substantial long-term functional dependence and imposes a heavy societal burden. However, although conventional functional training is a fundamental rehabilitation strategy, its efficacy is often limited by patients’ engagement levels, treatment costs, and the speed of treatment onset—issues that frequently lead to prolonged recovery periods and inconsistent therapeutic outcomes. To address these limitations, mounting evidence suggests that combining functional training with physical stimulation therapies can yield synergistic effects, thereby facilitating the functional recovery of stroke survivors (11, 13).

This study classifies the electromagnetic stimulation interventions included into two main categories: central nervous system stimulation techniques (rTMS, iTBS, tDCS, etc.); and peripheral nerve or muscle stimulation techniques (NMES, EA, TEAS, FES, etc.). Although there are marked differences between these stimulation modalities in terms of mechanisms of action, target sites, dosage parameters and clinical indications, they all share the common therapeutic principle of modulating neural excitability, promoting neural plasticity and improving motor dysfunction following stroke (20, 27, 28). Currently, there is a lack of direct head-to-head comparative studies between different electromagnetic stimulation protocols. To address this research gap, this study will employ a NMA approach to construct an analytical framework for comprehensively evaluating the efficacy and safety of the aforementioned neurostimulation techniques in treating lower limb dysfunction following stroke. Given that central and peripheral stimulation share consistent rehabilitation goals and similar mechanisms of neural plasticity regulation, it is clinically reasonable to incorporate these heterogeneous interventions into a single network meta-analysis framework; simultaneously, this study will conduct further subgroup analyses by central and peripheral stimulation to reduce inherent heterogeneity and enhance the interpretability of the pooled effect size results. By synthesising existing evidence-based data, this study will systematically evaluate the relative efficacy and safety of various published stimulation protocols for treating lower limb dysfunction following stroke. It aims to quantify differences in treatment efficacy across interventions and clarify their safety profiles, thereby providing high-level evidence-based support for the development of individualised and standardised rehabilitation strategies in clinical practice.
