# Supplementary material for: Comparing the effects of different electromagnetic stimulation on lower limb motor impairment after stroke: a protocol for systematic review and network meta-analysis
Source: Front Neurol. 2026 May 29;17:1828469. doi: 10.3389/fneur.2026.1828469 (PMC13259915; doi:10.3389/fneur.2026.1828469)
Supplement: Supplementary file 1 [file Data_Sheet_1.DOCX]

**Search strategy for the English databases.**

| **Order** | **strategy** |
| --- | --- |
| #1 | (Stroke[MeSH]) OR (Cerebrovascular Accident[Title/Abstract]) OR  (CVA[Title/Abstract]) OR (cerebral infarction[Title/Abstract]) OR (Brain Vascular  Accident[Title/Abstract]) OR (Apoplexy[Title/Abstract]) |
| #2 | (Hemiplegia[MeSH Terms]) OR (Paralysis[MeSH Terms]) OR (Motor function[Title/Abstract]) OR(Dysfunction [Title/Abstract]) OR (Lower limbs[Title/Abstract]) OR (Lower extremities[Title/Abstract]) OR(Leg[Title/Abstract]) OR (Digit[Title/Abstract]) OR(Toe [Title/Abstract]) OR (Knee [Title/Abstract]) OR(Ankle[Title/Abstract]) OR (Foot[Title/Abstract]) OR (Thigh[Title/Abstract]) OR (Lower limb[Title/Abstract])OR (Lower extremity[Title/Abstract]) |
| #3 | (Transcranial Magnetic Stimulation[MeSH]) OR (repetitive transcranial magnetic stimulation[Title/Abstract]) OR (rTMS[Title/Abstract]) OR (intermittent theta burst stimulation[Title/Abstract]) OR (iTBS[Title/Abstract]) OR (theta burst stimulation[Title/Abstract]) OR (Transcranial Direct Current Stimulation[MeSH]) OR (transcranial direct current stimulation[Title/Abstract]) OR (tDCS[Title/Abstract]) OR (Neuromuscular Electrical Stimulation[Title/Abstract]) OR (NMES[Title/Abstract]) OR (Functional Electrical Stimulation[Title/Abstract]) OR (FES[Title/Abstract]) OR (transcutaneous electrical acupuncture stimulation[Title/Abstract]) OR (TEAS[Title/Abstract]) OR (electroacupuncture[Title/Abstract]) OR (electro-acupuncture[Title/Abstract]) OR (Electric Stimulation Therapy[MeSH]) OR (electrical stimulation[Title/Abstract]) |
| #4 | (((((Randomized Controlled Trial[Title/Abstract]) OR (Controlled Clinical Trial[Title/Abstract])) OR (Random[Title/Abstract])) OR (Randomization[Title/Abstract])) OR (Random Allocation[Title/Abstract])) OR (Random[Title/Abstract]) |
| #5 | #1 AND #2 AND #3 AND #4 |

**Search strategy for the Chinese databases.**

| **Order** | **strategy** |
| --- | --- |
| #1 | SU=('电刺激'+'电疗'+'神经电刺激'+'肌肉电刺激'+'神经肌肉电刺激'+'功能性电刺激'+'刺激电极'+'经皮电刺激'+'经皮穴位电刺激'+'经皮神经电刺激'+'电针'+'中频电疗仪'+'经颅磁刺激'+'磁刺激'+'非侵入性脑刺激'+'经颅神经刺激'+'经颅直流电刺激'+'表面神经肌肉电刺激'+'感觉神经肌肉电刺激'+'间歇性θ爆发刺激'+'穴位电刺激'+'θ波爆发刺激'+'非侵入性脑刺激'+'重复经颅磁刺激'+'神经介导的血管舒张刺激'+'NIBS'+'sNMES'+'NMES' +'tDCS' +'TBS' +'TBS' +'rTMS') |
| #2 | SU=('中风'+'脑卒中'+'卒中'+'脑梗死'+'脑梗塞'+'脑缺血'+'脑出血'+'脑血管病'+'缺血性卒中'+'出血性卒中') |
| #3 | SU=('重复经颅磁刺激'+'rTMS'+'经颅直流电刺激'+' tDCS'+'功能性电刺激'+'FES'+'神经肌肉电刺激'+'NMES'+'经皮穴位电刺激'+'TEAS'+'电针') |
| #4 | SU=('下肢功能障碍'+'下肢偏瘫'+'下肢活动不利'+'下肢偏瘫'+'下肢') |
| #5 | #1 AND #2 AND #3 AND #4 |
